# Supplementary material for: Does structural social capital lead to proactive green innovation? a three-part serial mediation model
Source: PLoS One. 2024 Apr 5;19(4):e0301286. doi: 10.1371/journal.pone.0301286 (PMC10997068; doi:10.1371/journal.pone.0301286)
Supplement: S1 Table — (DOCX) [file pone.0301286.s001.docx]

**S1 Table. Questionnaire items**

| Construct | From 1 – strongly disagree – to 7 – strongly agree |
| --- | --- |
|  | Structural Social Capital (SSC) |
| SSC1 | Acquires knowledge from our inter-organizational contacts’ network |
| SSC2 | Personally meets contacts who acquire external knowledge |
| SSC3 | Maintains close inter-relationships with contacts who acquire external knowledge. |
| SSC4 | Maintains frequent inter-relationships with contacts who acquire external knowledge |
| SSC5 | (In general) Has contacts who acquire knowledge from among themselves |
|  | Cognitive Social Capital (CSC) |
| CSC1 | Shares goals and projects interests with its external relationships |
| CSC2 | Shares language and a common vision regarding the functioning and factors of success of the environment with external agents (relationships) |
| CSC3 | Understands work techniques in a similar way to the external agents with whom it has relationships |
| CSC4 | Shares a common culture with external agents from repeated interactions |
|  | Relational Social Capital (RSC) |
| RSC1 | Employees in cross-organizational cooperation in feel confidence on other employees’ skills and abilities to do their work |
| RSC2 | Employees in cross-organizational cooperation have relationships based on reciprocal faith and respect. |
| RSC3 | Employees in cross-organizational cooperation trust on other employees’ intentions and behaviors. |
| RSC4 | Employees in cross-organizational cooperation have reciprocal faith in others behaviors to work towards organizational best interest/goals. |
| RSC5 | Employees in cross-organizational cooperation trust and esteem one another even if they are not close friends. |
|  | Dynamic Capability (DC) |
| DC1 | In my organization people participate in professional association activities. |
| DC2 | In my organization we use established processes to identify target market segments, changing customer needs and customer innovation. |
| DC3 | In my organization we observe best practices in our sector. |
| DC4 | In my organization we gather economic information on our operations and operational environment. |
| DC5 | In my organization we invest in finding solutions for our customers. |
| DC6 | In my organization we adopt the best practices in our sector. |
| DC7 | In my organization we respond to defects pointed out by employees. |
| DC8 | In my organization we change our practices when customer feedback gives us a reason to change. |
| DC9 | In recent years, we have carried out the implementation of new kinds of management methods. |
| DC10 | In recent years, we have carried out the new or substantially changed marketing method or strategy. |
| DC11 | In recent years, we have carried out the Substantial renewal of business processes |
| DC12 | In recent years, we have carried out the new or substantially changed ways of achieving our targets and objectives. |
|  | Proactive Green Innovation (PGI) |
| PGI1 | The company frequently engages in proactive environmentally-related innovation activities to gain a competitive edge in product offerings. |
| PGI2 | The company is committed to consistently investing capital in green innovation to seize opportunities and lead the market. |
| PGI3 | The company actively enhances its production processes to reduce costs through methods like reusing and recycling. |
| PGI4 | The company spontaneously initiates innovative approaches related to the environment to gain a competitive advantage. |
